# Quantitative Cross-Platform Performance Comparison between Different Detection Mechanisms in Surface Plasmon Sensors for Voltage Sensing

**DOI:** 10.3390/s18093136

**Published:** 2018-09-17

**Authors:** Phitsini Suvarnaphaet, Suejit Pechprasarn

**Affiliations:** College of Biomedical Engineering, Rangsit University, Pathum Thani 12000, Thailand; phitsini.s@rsu.ac.th

**Keywords:** surface plasmon resonance, plasmonic measurement mechanism, voltage sensing, instrumentation

## Abstract

Surface plasmon Resonance (SPR) has recently been of interest for label-free voltage sensing. Several SPR structures have been proposed. However, making a quantitative cross-platform comparison for these structures is not straightforward due to (1) different SPR measurement mechanisms; (2) different electrolytic solution and concentration in the measurement; and (3) different levels of external applied potential. Here, we propose a quantitative approach to make a direct quantitative comparison across different SPR structures, different electrolytic solutions and different SPR measurement mechanisms. There are two structures employed as example in this theoretical study including uniform plasmonic gold sensor and bimetallic layered structure consisting of uniform silver layer (Ag) coated by uniform gold layer (Ag). The cross-platform comparison was carried by several performance parameters including sensitivity (S), full width half maximum (*FWHM*) and figure of merit (*FoM*). We also discuss how the SPR measurement mechanisms enhance the performance parameters and how the bimetallic layer can be employed to enhance the *FoM* by a factor of 1.34 to 25 depending on the SPR detection mechanism.

## 1. Introduction

Voltage sensing has been a crucial tool used to study important biological processes, such as, neurotransmitter signaling [1,2,3], cell membrane voltage sensing [4] and electrical signals in cardiac cells [5], muscles [6] and skin tissues [7]. Scientists and engineers have employed and developed several optical-based techniques to measure electrophysiological potentials in cells and cell membranes including the use of fluorescence dyes [8,9] and interferometric detection through microfiber [10,11]. However, there are some issues that still need to be addressed. Although the fluorescence dyes can be combined with microscopy techniques allowing sub-cellular activities to be imaged, they however might affect the nature of the biological samples and obscure the accuracy of the voltage measurement [12]. The fluorescence labelling also suffers from photobleaching, making a long run experiment not practical [13]. The microfiber approach requires the microfiber to be placed very close to the sample, making the optical alignment difficult and the microfiber also obscures the sample accessibility [14].

**Figure 1:**
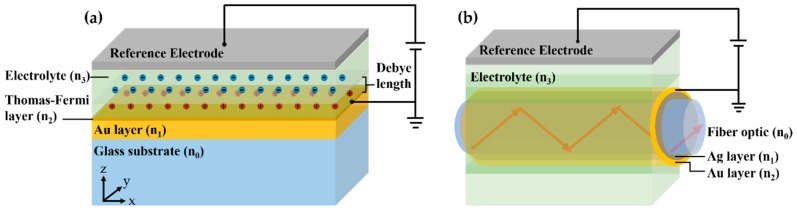
Illustration of (**a**) a uniform plasmonic gold sample; (**b**) bimetallic layers of gold (Au) and silver (Ag) wrapping around the fiber optic core.

Recently, there has been an interest in employing label-free detection using surface plasmon resonance (SPR) as a voltage sensing platform for biological samples [15,16,17,18,19]. The SPR has been a gold standard for real-time binding kinetics studies [20] and very low concentration biological measurements [21]. The theory and applications for SPR in biosensing have been very well established. However, the application of SPR in voltage sensing has not been reported much in the literature. Abayzeed et al. [17] proposed a model based on the Drude model [22] to calculate the effect of charge potential on the electron density and refractive index in Thomas-Fermi length of uniform plasmonic gold layer as shown in Figure 1a. They have also experimentally validated and found that their experimental results agree very well with the model. Huang et al. [18] proposed a bimetallic layer consisting of a silver layer coated by a gold layer wrapping around a fiber optic core as shown in Figure 1b. Zhang et al. [23] proposed a periodic array of plasmonic nanorods reporting an ultra-sensitive voltage detection. However, there is no obvious way to make a direct performance comparison between these structures, since the structures used (1) different types of electrolytes and concentrations, as well as (2) different optical detection mechanisms and incident wavelengths.

In this paper, we develop and propose a theoretical framework to evaluate and compare the performance of a uniform plasmonic gold sensor and bimetallic layer for voltage sensing using Fresnel equations. These two structures were employed as an example for the proposed procedure. It is noted that the proposed concept can also be applied to some other SPR structures and biosensing in general. To the best of the authors’ knowledge, this has never been reported in the literature before. We also discuss a feasible bimetallic structure, which enhanced the sensitivity and the *FoM* by a factor of 1.34 to 25 as discussed in detail in the later section.

## 2. Materials and Methods

### 2.1. Effect of External Applied Voltage on the Refractive Index of Plasmonic Metal

#### 2.1.1. Gouy-Chapman-Stern Model

**Figure 2:**
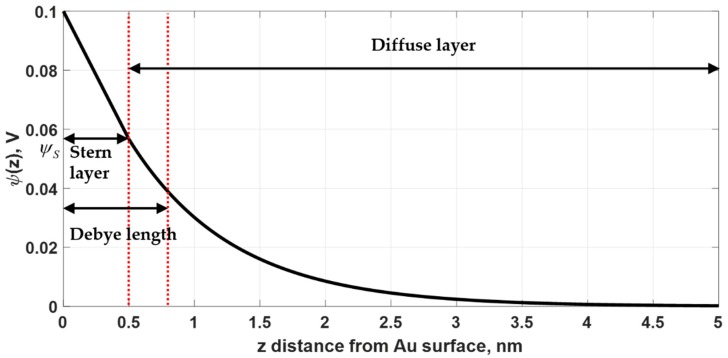
Voltage potential profile using Gouy-Chapman-Stern model. The Stern potential was calculated for the applied voltage $\psi_{0}$ of 0.1 V and the electrolyte was 0.15 M NaCl solution.

In this paper, we follow a model developed by Abayzeed et al. [17]. The optical properties of the metal are modelled by a double layer capacitor. When external voltage is applied to the SPR metal sensor, there are charges confined at the interface between the electrolyte and the metal surface as shown Figure 1a. These charges are confined in Debye length, which is defined as the distance from the sensor surface that the applied potential is dropped to 1/*e* level [24] as shown in Figure 2. The interfacial potential can be calculated for different electrolytes and voltage potentials using Gouy-Chapman-Stern model [24]. The model considers the applied voltage potential, electrolyte concentration, number of valence ions, ion density and optical permittivity of the electrolyte. The voltage potential is a function of distance from the metal surface *z* and is given by:

(1) $$\psi \left(z\right)=\psi_{0}-z\sqrt{\frac{8k_{B}TN_{0}}{\varepsilon_{0}\varepsilon }}\sinh\left(\frac{\zeta e\psi_{S}}{2k_{B}T}\right)$$

where

ψ(z) is the voltage potential as a function of distance from the metal surface, in volt (V).
$\psi_{0}$ is the applied voltage on the metal surface, in volt (V).
z is the distance from the metal surface towards the electrolyte, in meter (m).
$k_{B}$ is the Boltzmann constant, $k_{B}$ = 1.3806 × 10^−23^ m^2^kg s^−2^K^−1^.
T is the temperature of the electrolyte in K.

In this paper, the temperature has been fixed to 25 °C or 298.15 K. The term $k_{B}T$ is the thermal energy 4.1162 × 10^21^ J. $N_{0}$ is the ion density, which can be calculated by concentration in molar [c] in mol/dm^3^ of the electrolyte solution times Avogadro’s number $A_{V}$ of 6.0221 × 10^23^ and 1000 dm^3^ conversion factor to the SI unit or $N_{0}=1000[c]A_{V}\cdot \zeta$ is the valence of the ion. In this paper, all the cases were simulated with 0.15 M NaCl electrolyte solution. This molar concentration is equivalent to 0.9% concentration of NaCl solution. The valence of the ion for NaCl is 1 [1]. $\varepsilon_{0}$ is the free space permittivity 8.8542 × 10^−12^ F/m and ε is the static relative permittivity of the solute, which is water in this case. The ε for water is 78.54 at 25 °C [2]. e is the electron elementary charge of 1.6022 × 10^−19^ C. $\psi_{S}$ is Stern potential, which can be calculated as the potential level from Equation (1) at the solvent molecule size, $Z_{S}$. The $Z_{S}$ for NaCl molecule is 564.02 pm or 0.5640 nm [3]. By substituting these parameters into Equation (1) and solving the equation, the corresponding Stern potential is 0.0564 V for $\psi_{0}$ of 0.1 V as shown in Figure 2.

#### 2.1.2. Capacitor Model

The applied voltage $\psi_{0}$ also affects the free electron density in the metal as depicted in Figure 1a. There is an accumulation or depletion of free electrons in the metal in Thomas-Fermi screening length, *d_TF_*. Gold has been chosen as the plasmonic material in this study, since gold is chemically stable and is not toxic to the biological samples [25,26,27]. The *d_TF_* for gold is 0.5 Å [28]. These two layers, namely Debye layer and Thomas-Fermi layer can then form a double layer capacitor, whose capacitance can be calculated as [23]:

(2) $$\frac{1}{C}=\frac{z_{S}}{\varepsilon_{0}\varepsilon }+\frac{1}{\sqrt{\frac{2\varepsilon_{0}\varepsilon N_{0}\zeta^{2}e^{2}}{k_{B}T}}\cosh\left(\frac{\zeta e\psi_{S}}{2k_{B}T}\right)}$$

where C is the capacitance in F of the double layer capacitor. The rest of the variables are the same as described in Equation (1). The capacitance for the applied voltage $\psi_{0}$ of 0.1 V and the 0.15 M NaCl electrolytic solution is 0.7158 F calculated from Equation (2).

#### 2.1.3. Change in Charge Density Due to the Applied Voltage

Once the capacitance is determined, we can then determine the change in free electron density of the plasmonic metal, $\Delta N_{e}$ using the McIntyre model [4], which is given by:

(3) $$\Delta N_{e}=\frac{C\psi_{0}}{-ed_{TF}}$$

where $\Delta N_{e}$ is the change in free electron density in the plasmonic metal. The plasma frequency $\omega_{p}$ can be then determined from the plasma frequency equation given by:

(4) $$\omega_{p}=\sqrt{\frac{(N+\Delta N_{e})e^{2}}{m_{e}\varepsilon_{0}}}$$

where $\omega_{p}$ is the plasma frequency in rad/s. *N* is the electron density, which is 5.90 × 10^28^ electrons/m^3^ [5]. *m_e_* is the electron mass, which is 9.1094 × 10^−31^ kg.

#### 2.1.4. Drude Model

The complex permittivity in the Thomas-Fermi screening length $\varepsilon_{TF}$ can be calculated from the Drude model [6], which is expressed as:

(5) $$\varepsilon_{TF}(\omega )=1-f_{0}\frac{\omega_{p}^{2}}{\omega (\omega +\Gamma_{0}i)}$$

where

ω is the angular frequency of the incident light in eV.
$\varepsilon_{TF}$ is the complex permittivity in the Thomas-Fermi screening length.
$f_{0}$ is the oscillator strength, which is 0.760 eV for gold [6].
$\Gamma_{0}$ is the damping constant, which is 0.053 eV for gold [6].

The refractive index of the Thomas-Fermi screening length $n_{TF}$ can be calculated by taking the square root of Equation (5).

This external applied voltage moderates the Thomas-Fermi layer as explained in the models and the gold layer refractive index in the *d_TF_* is then modulated by the applied voltage. This consequently reflects in plasmonic shift as shown in the next section.

### 2.2. Simulation Models

#### 2.2.1. Simulation Model for Uniform Gold Sample

**Figure 3:**
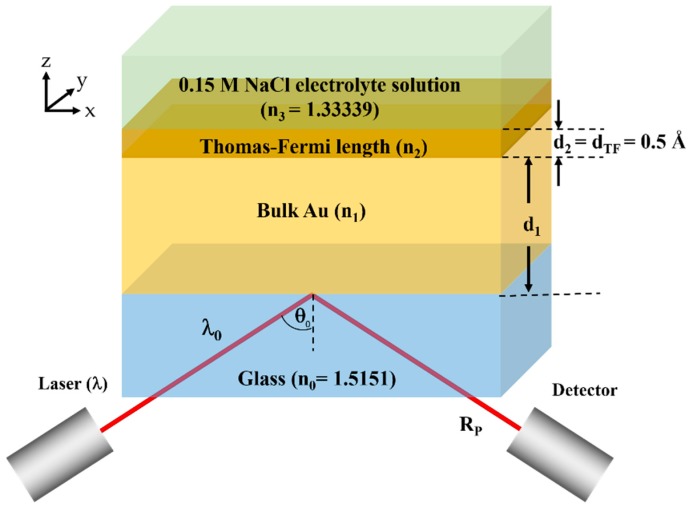
Simplified model for uniform gold SPR sensor consisting of the charged Thomas-Fermi screening length of 0.5 Å and the uniform bulk gold layer.

The plasmonic gold layer in this study is modelled by two gold layers as shown in Figure 3. The first layer of gold is the bulk gold layer with thickness d_1_ and refractive index *n*_1_. The refractive indices of the bulk gold layer for each wavelength is extracted from Johnson and Christy 1972 [7]. The second gold layer is the Thomas-Fermi layer with the refractive index *n*_2_ and thickness *d*_2_ of 0.5 Å. The refractive index of the Thomas-Fermi layer is determined as described in Equation (5) in the earlier section. The p-polarized incident beam with the free-space incident wavelength of λ and incident angle of $\theta_{0}$ is incident on this plasmonic sensor through a glass substrate with refractive index *n*_0_ of 1.5151 (BK7) [8]. Reflectance of the p-polarized light *R_p_* is then calculated using Fresnel equations through the transfer matrix approach [9].

#### 2.2.2. Simulation Model for Bimetallic Sample

**Figure 4:**
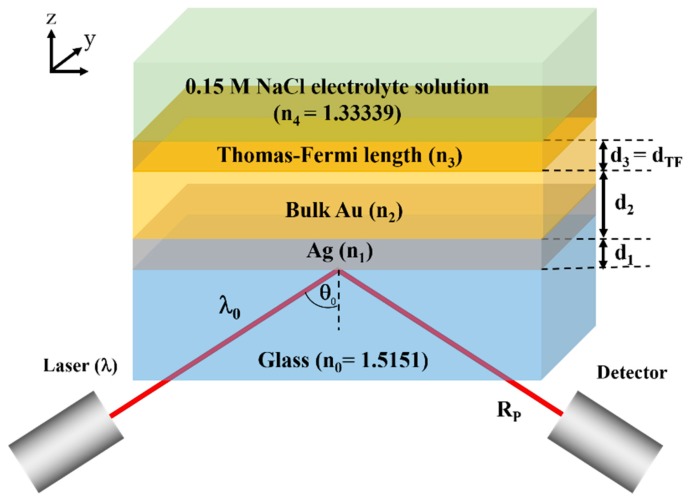
Simplified model for bimetallic SPR sensor consisting of a uniform silver layer and two gold layers, which consists of a uniform bulk gold layer and the top gold layer is the charged Thomas-Fermi screening length of 0.5 Å.

Bimetallic plasmonic sensor for voltage sensing consisting of a uniform silver layer coated by another uniform gold layer as shown in Figure 4 were employed in this study. The simulation concept explained in the above section is also applied for this case. There is a silver layer with a thickness of *d*_1_ and a refractive index *n*_1_ extracted from Johnson and Christy 1972 [7] coated on the top of the BK7 glass substrate. The silver layer is then coated by a uniform gold layer. In the simulation, the gold layer is simulated using two sublayers, which is firstly the bulk gold layer with a thickness of *d*_2_ and the refractive index of *n*_2_ extracted from Johnson and Christy 1972 [7], and the Thomas-Fermi length layer with the thickness of *d*_3_ of 0.5 Å and refractive index calculated by the models explained in Section 1.

#### 2.2.3. Voltage Range in this Study

It is important to point out that this model is only valid for the applied potential range between −200 mV to 200 mV. The external potential outside this range could lead to a chemical reaction on the surface of the gold and the shape of the SPR dip can be dramatically distorted as reported by Garland et al. [10]. The voltage range in this study was −200 mV to 200 mV, which was in the typical range of typical electrochemical processes in biological samples [11].

#### 2.2.4. Performance Comparison

**Figure 5:**
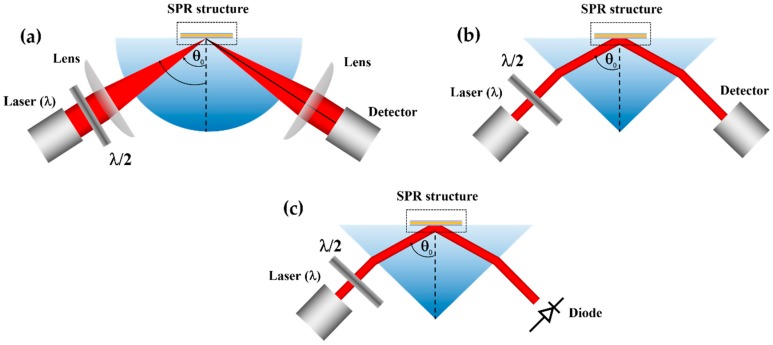
Illustration of SPR sensing structure based on Kretschmann configurations for (**a**) *M1*, (**b**) *M2*, and (**c**) *M3*.

In SPR measurement, there are several approaches to measure the change in SPR signal including intensity measurement [12] and phase measurement [13]. In this paper, we chose three common practice approaches for SPR measurement which were:

Mechanism 1 (*M1*) measures the change in plasmonic coupling angle $\theta_{SP}$ at a fixed incident wavelength λ as shown in Figure 5a.

Mechanism 2 (*M2*) measures the change in plasmonic coupling wavelength $\lambda_{SP}$ at a fixed incident angle $\theta_{0}$ as shown in Figure 5b.

Mechanism 3 (*M3*) measures the change in reflectance intensity at a fixed incident angle $\theta_{0}$ a fixed incident wavelength λ as shown in Figure 5c.

**Figure 6:**
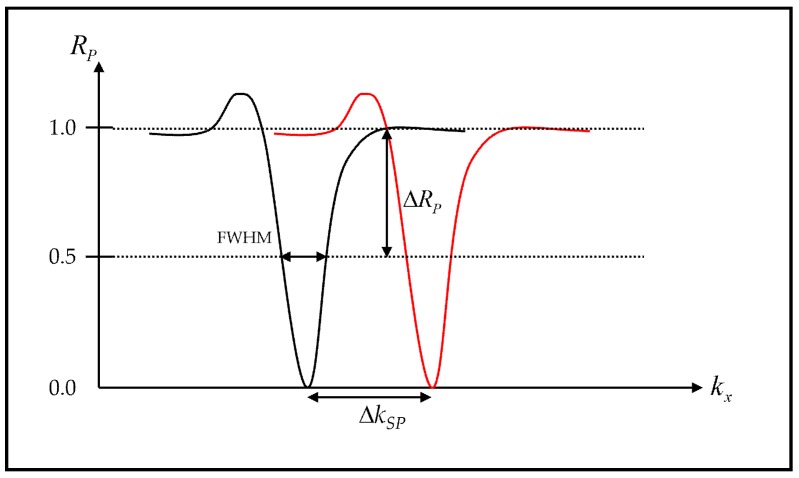
Illustrations showing how the parameters were measured. *R_p_* response for $\psi_{0}$ of 0 V (solid black curve) and *R_p_* response for $\psi_{0}$ greater than 0 V (solid red curve).

In this paper, we would like to quantify and compare different structures and different detection mechanisms. Let us define some terms to quantify the performance as shown in Figure 6.

#### 2.2.5. Figure of Merit for *M1* and *M2*

The Figure of Merit (*FoM*) is the term indicating the performance of a sensor. The *FoM* is defined here as a ratio between sensitivity and the full width half maximum. The *FoM* term takes into account (1) how much the SPR dip moves and (2) how narrow the SPR dip is. This has been widely accepted as a standard performance parameter for biosensors [14].

(6) $$FoM_{M1,M2}=\frac{S}{FWHM}$$

The wave vector of the incident light along *x*-axis, *k_inc,x_* is defined as $\frac{2\pi n_{0}}{\lambda }\sin\theta_{0}$ and the wave vector of the surface plasmons is defined as $\frac{2\pi n_{0}}{\lambda_{sp}}\sin\theta_{sp}$. The sensitivity (*S*) for *M1* and *M2* is defined as:

(7) $$S_{M1,M2}=\frac{\Delta k_{sp}}{\Delta \psi_{0}}$$

where

*S* is the sensitivity term in m^−1^V^−1^.
$\Delta k_{sp}$ is the change in plasmonic wave vector.
$\Delta \psi_{0}$ is the change in applied potential.

The *FWHM* is defined as the width in wave vector space that the reflectance intensity is less than 0.5.

#### 2.2.6. Figure of Merit for *M3*

The *FoM* for the *M3* case is defined as:

(8) $$FoM_{M3}=\alpha \frac{S_{M3}}{R_{p,0V}}$$

where $S_{M3}$ is the sensitivity of the third detection mechanism, which is defined as $\frac{\Delta R_{p}}{\Delta \psi_{0}}$. The term $\Delta R_{p}$ is the change in reflectance intensity, the term $R_{p,0V}$ is the reflectance intensity level when $\psi_{0}$ of 0 V is applied, and α is the normalization factor allowing this *M3 FoM* to be compared with *M1* and *M2 FoMs*. The value of α is 1.5741 × 10^−6^. The derivation of the normalization factor will be explained in the next section.

## 3. Results and Discussion

### 3.1. Uniform Gold Sample for Voltage Sensing

#### 3.1.1. Measurement Mechanism 1 (*M1*)

**Figure 7:**
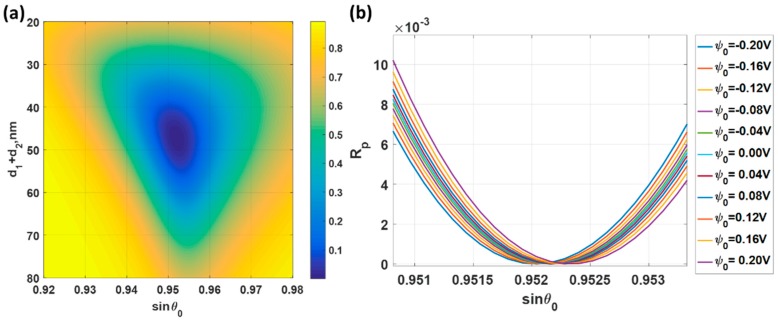
(**a**) *R_p_* responses for different incident angles ranging from 0.92 to 0.98. The applied potential $\psi_{0}$ of 0 V was applied at the incident wavelength λ of 632.8 nm; (**b**) *R_p_* responses when different voltage levels were applied. The responses were simulated with the total gold thickness of 47 nm and the incident wavelength λ of 632.8 nm.

**Figure 8:**
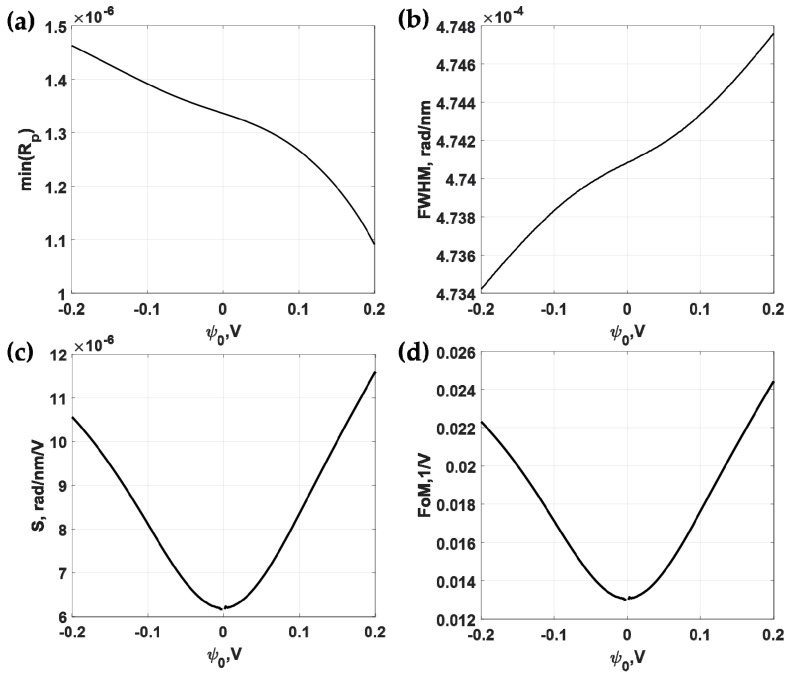
Responses of the 47 nm thick uniform gold sensor for different applied potential levels ranging from −0.2 V to 0.2 V and the incident wavelength λ of 632.8 nm includes (**a**) minimum reflectance intensity level at the plasmonic angles min (*R_p_*); (**b**) full width half maximum; (**c**) sensitivity; and (**d**) Figure of Merit.

Firstly, let us consider the uniform gold sensor as shown in Figure 3 as a basis to compare the other types of structures. Figure 7a shows reflectance responses of the p-polarized incident light at 632.8 nm (HeNe laser wavelength) incident wavelength when the total gold thickness was varied from 20 nm to 80 nm and the applied potential was $\psi_{0}$ of 0 V. The total gold thickness that gave the minimum reflectance dip was 47 nm, which was in the range of optimum gold thickness for SPR sensor [15]. This 47 nm gold sensor was then calculated for different applied voltage levels as shown in Figure 7b. The responses here agree very well with the results reported in Abayzeed et al. [16]. The results in Figure 7b allows us to calculate the defined performance parameters as shown in Figure 8. Figure 8a shows the minimum reflectance intensity level. It can be seen from the figure that the depth of the dip was not affected much by the range of applied voltage level maintaining a decent SPR reflectance dip response over the whole applied voltage range. The *FWHM* was also not affected much by the applied voltage level as shown in Figure 8b. The variation in the *FWHM* was within 0.15%. The sensitivity (*S*) was slightly different for positive and negative potentials and the maximum sensitivity was 1.16 × 10^−5^ rad/nm/V at $\psi_{0}$ of 0.2 V as shown in Figure 8c. The maximum *FoM* of 0.0244 V^−1^ at applied potential $\psi_{0}$ of 0.2 V ss shown in Figure 8d.

Another approach to compare this sensitivity is to compare it with bulk refractive index sensitivity as if this 47 nm was employed as a conventional SPR sensor to measure the change in the refractive index of the sample [14]. In other words, *n*_1_ and *n*_2_ were identical and the refractive index of *n*_3_ were varied until the change in $k_{SP}$ or $\Delta k_{SP}$ of 1.16 × 10^−5^ rad/nm was reached. The change in *n*_3_ refractive index required to change the *k_sp_* by 1.16 × 10^−5^ rad/nm was 2 × 10^−4^ RIU. This number does give us some idea of the voltage detection limit of the uniform gold sensor. The current state of the art angular-based SPR platform relying on phase detection can achieve around 10^−6^ to 10^−7^ RIU detection limit [17]. The intensity detection mechanism, of course, has a lower RIU detection limit around 10^−5^ to 10^−6^ RIU [12]. This indicates the voltage detection limit of the uniform gold sensor is around 50 mV for 10^−5^ RIU detection limit, which is not very good. Therefore, another type of SPR sensor and detection mechanism was also developed and reported by several authors [18,19].

#### 3.1.2. Measurement Mechanism 2 (*M2*)

**Figure 9:**
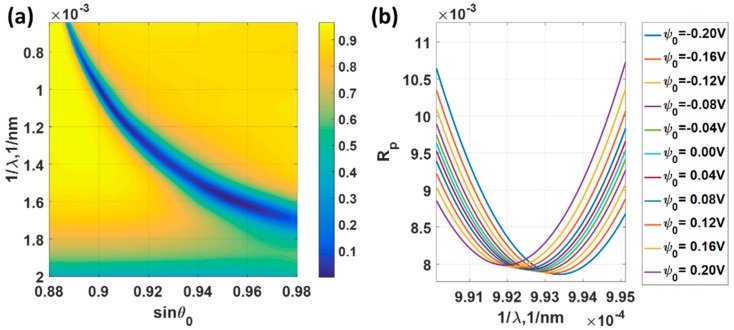
(**a**) Reflectance spectra of different incident angles ranging from 0.88 to 0.98 and incident wavelengths ranging from 500 nm to 1550 nm, when the 47 nm gold sensor was biased with $\psi_{0}$ of 0 V. (**b**) Reflectance spectra of 47 nm gold sensor for different applied potentials when the incident angle was fixed at sin*θ*_0_ of 0.9.

Results for wavelength scanning systems in optics are usually expressed in incident wavelength, λ. Here, the results for wavelength scanning were expressed as 1/λ, which was a linear function of the wave vector. We will show later that by expressing the results in wave vector, this allowed us to compare the performance across different detection mechanisms. The gold thickness of 47 nm has also been employed in this analysis and the refractive indies of the gold for each incident wavelength λ were extracted from Johnson and Christy 1972 [7]. Figure 9a shows reflectance spectra of different incident angles and incident wavelengths, when the 47 nm gold sensor was biased with $\psi_{0}$ of 0 V. The lower operating angle gives a narrow full width half maximum spectra. Figure 9b shows reflectance spectra of 47 nm gold sensor for different applied potentials when the incident angle was fixed at sin*θ*_0_ of 0.9. The responses like Figure 9b were also calculated for sin*θ*_0_ of 0.92, 0.94 and 0.96, but they are omitted to shorten down the length of the manuscript.

**Figure 10:**
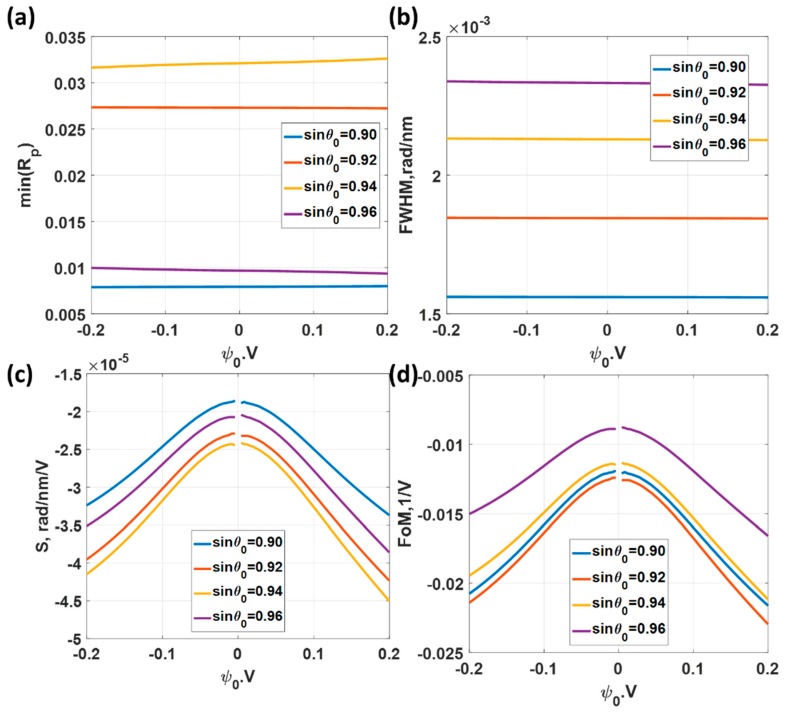
Responses of the 47 nm thick uniform gold sensor for different applied potential levels ranging from −0.2 V to 0.2 V and the incident angles of sin*θ*_0_ of 0.90, 0.92, 0.94 and 0.96. (**a**) Minimum reflectance intensity level at the plasmonic wavelengths min(*R_p_*); (**b**) full width half maximum; (**c**) Sensitivity; and (**d**) Figure of Merit.

Figure 10a shows the minimum reflectance levels for different incident angle sin*θ*_0_ varying from 0.90 to 0.96. The minimum reflectance for all the cases was less than 3.5% level, indicating that the ranges can provide a decent SPR dip response. However, the *FWHM* does vary by the operating angle as shown in Figure 10b. The lower operating angle sin*θ*_0_ has a lower *FWHM* compared to bigger operating angle cases. The *FWHM* of the sin*θ*_0_ of 0.90 case was 1.5 times narrower than the sin*θ*_0_ of 0.96 case. It is interesting to point out that the sensitivity was not the same for all cases and did not vary linearly by the operating angle sin*θ*_0_ position. The highest sensitivity of 4.51 × 10^−5^ rad/nm/V occurred at sin*θ*_0_ of 0.94 and the lowest sensitivity of 3.37 × 10^−5^ rad/nm/V was the operating angle of sin*θ*_0_ of 0.90. Although the *FWHM* for the sin*θ*_0_ of 0.90 came up as the best performance, the sensitivity for the sin*θ*_0_ of 0.90 appeared to be the worst in this simulation set. Since the same performance parameters were used in *M1* and *M2*, this allows us to make a direct comparison between the two sensing mechanisms. The sensitivity for this wavelength scanning system was much higher than the angular scanning system by almost a factor of four, as shown in Figure 10c in comparison with Figure 8c. On the one hand, the *FWHM* for the wavelength scanning cases were almost 3 to 4 times wider than the *FWHM* for the angular scanning case as shown in Figure 10b in comparison with Figure 8b. This superior sensitivity for the wavelength scanning system was then cancelled out by the *FWHM* in the *FoM*. The *FoM* of 0.0230 V^−1^ for the wavelength scanning was slightly worse than the *FoM* of 0.0244 V^−1^ of angular scanning case as shown in Figure 10d in comparison with Figure 8d. The *FoM* in Figure 10d indicates that it is better to setup the SPR platform at a lower incident angle for the wavelength scanning system. Note that the negative values of the sensitivity and the *FoM* indicate that the SPR dip movement moved in the opposite direction to the *M1*. For *M1* the $k_{SP}$ increased as the applied voltage increased; whereas for *M2*, the $k_{SP}$ decreased as the applied voltage increased.

It is worth pointing out that some of the measurement mechanisms do not rely on the intensity of the dip, but rely mostly on how much the dip moves, such as interferometric detection [14]. If these techniques were implemented, it is therefore better to align the optical system for wavelength coupling measurement since it has the higher sensitivity compared to angular measurement.

#### 3.1.3. Measurement Mechanism 3 (*M3*)

**Figure 11:**
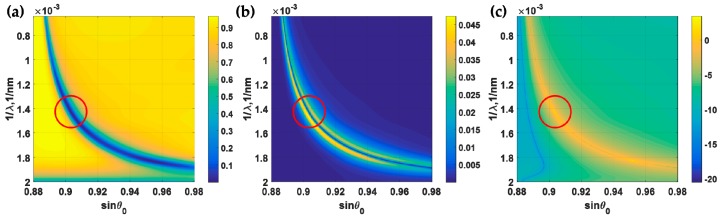
(**a**) Reflectance spectra of different incident angles and incident wavelengths, when the 47 nm gold sensor was biased with *ψ*_0_ of 0.2 V. (**b**) Sensitivity for *M3* calculated using Figure 9a and Figure 11a,c. (**c**) *FoM*, calculated using Equation (8). Red arced circles highlight the maximum *FoM*.

In this section, the optimum thickness of 47 nm is also employed. To calculate the *FoM* for *M3*, a set of calculations for the same incident wavelengths and angles as shown in Figure 9a were calculated; however, this time with the applied voltage $\psi_{0}$ of 0.2 V. The reflectance spectra shown in Figure 11a allow us to calculate the sensitivity as shown in Figure 11b. The *FoM* was then calculated by Equation (8) and is shown in Figure 11c. Note that the *FoM* in Figure 11c is shown in logarithmic base 10 scale for clarity. The maximum *FoM* of 2735 V^−1^ occurred at the incident angle sin*θ*_0_ of 0.90 and incident wavelength λ of 698.8 nm as highlighted by the arced circle in Figure 11. We will later see that these *FoM* values can be enhanced further by the bimetallic layer. Table 1 summarizes the performance parameters for the discussed uniform gold SPR sensors.

Figure 11b shows us that the maximum sensitivity was around 0.0474. This occurred at sin*θ*_0_ of 0.9042 and λ of 898.5 nm. This operating position had the minimum *R_p_* of 0.2659. This sensitivity and this *R_p_* gave 0.1783 S/*R_p_* ratio. The *S*/*R_p_* is sensitivity per input power. This was a lot lower than the *S*/*R_p_* of the operating points shown in Table 1, *M3*. Nowadays, there are a wide variety of high power light sources, however, the fundamental sensitivity per unit power cannot be changed. This is the reason why we defined the performance parameter as sensitivity per unit power.

The *S*/*R_p_* cannot be directly compared with the *FoM* for *M1* and *M2*. The *FoM* performance for *M1* and *M2* were very similarly around 0.023 to 0.024. Under the condition that *FoM* performance for angular scanning and wavelength scanning were very similar, it has enabled us to confidently believe that the *FoM* for *M3* mechanism should give a similar *FoM* response, since for *M3* the incident angle and wavelength were fixed. The normalization factor, α, was then introduced by normalizing the *FoM* of *M3* to 0.0244 value by a normalization factor, α 0.0244/15,500 or 1.5742 × 10^−6^.

### 3.2. Bimetallic Layer for Voltage Sensing

Huang et al. [20] proposed a bimetallic layered structure consisting of a silver layer coated by a uniform gold layer wrapping around an optical fiber core. It is important to point out that the bimetallic layer structure proposed by Huang et al. [20] was a bimetallic layer coated on an optical fiber core. The sensitivity depended on the amount of transmission through the fiber, in other words, the SPR detection mechanism was the amount of light absorption in the metal. In this section, we consider only the thin film bimetallic structure as shown in Figure 4. The thin film is applicable for in vitro SPR microscopic sensing [15,21,22,23,24]. Here, the gold layer was in contact with the 0.15 M NaCl electrolytic solution, since the gold is chemically stable and inactive [25,26] whereas silver is highly reactive and can also form hazardous substances [27], such as, silver sulfide Ag_2_S. The thicknesses of the silver and gold layers considered in this paper were at least 15 nm thick due to two major reasons. Firstly, a very thin layer of gold and silver usually form some defects and islands [28]. The second issue is that all the simulations presented in this manuscript the plasmonic materials were treated as bulk gold. There are several papers reported that there are abnormal transmission spectra when the gold layer and silver layer are thinner than 15 nm [29,30].

#### 3.2.1. Measurement Mechanism 1 (*M1*)

**Figure 12:**
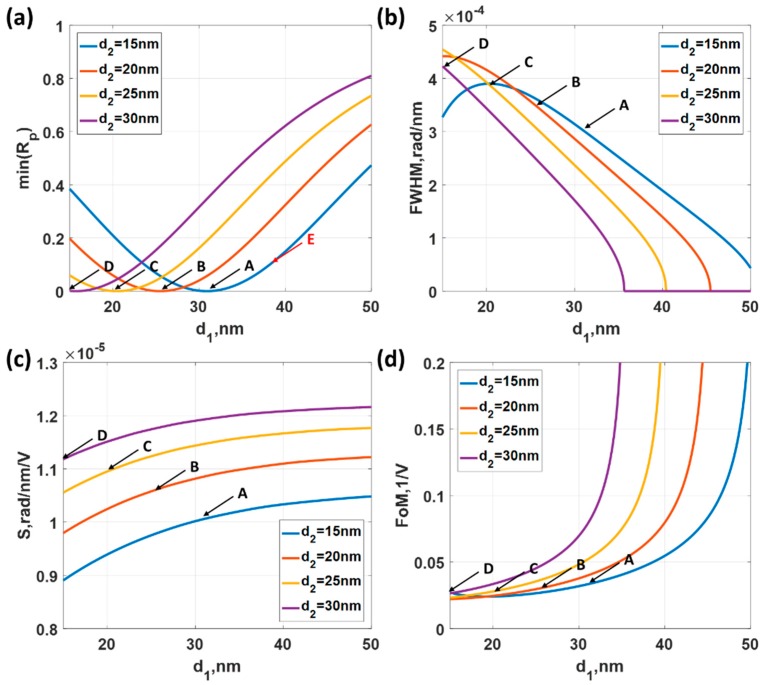
Performance parameters calculated by varying silver layer thickness *d*_1_ from 15 nm to 50 nm and gold thickness *d*_2_ of 15 nm (solid blue curve), 20 nm (solid red curve), 25 nm (solid yellow curve) and 30 nm (solid purple curve) when the incident wavelength λ was fixed at 632.8 nm and the applied potential *ψ*_0_ of 0 V at (**a**) minimum *R_p_*; (**b**) *FWHM*. (**c**) Sensitivity calculated by comparing the plasmonic wave vector positions of applied potentials *ψ*_0_ of 0 V and 0.2 V and (**d**) *FoM* calculated from Figure 12b,c.

Figure 12a shows the minimum intensity of the reflectance spectra when the thickness of silver *d*_1_ was varied from 15 nm to 50 nm and the thickness of gold *d*_2_ was 15 nm, 20 nm, 25 nm and 30 nm. The minimum reflectance occurred when the total thickness of *d*_1_ and *d*_2_ was around 46 nm as labelled by ‘A’, ‘B’, ‘C’ and ‘D’ in Figure 12a. The *FWHM* for the silver layer is usually narrower than the gold layer, the bilayer structure with thicker layer of silver compared to the gold layer had a lower *FWHM* as shown in Figure 12b. The label ‘A’ had the narrowest *FWHM* followed by ‘B’, ‘C’ and ‘D’, respectively. However, the sensitivity had an opposite trend to the *FWHM*. The narrowest *FWHM* had the lowest sensitivity and the worst *FWHM* had the best sensitivity as shown in Figure 12c. The opposite trend of *FWHM* and the sensitivity, of course, cancelled out in the *FoM*.

It is important to point out that if the sensitivity or how far the plasmonic dip moves is more important than the depth of the SPR dip. This is the case for the confocal surface plasmon microscopy system [15,21,22] and SPR phase measurements [13,31]. It is then better to operate at slightly higher reflectance intensity as labelled in ‘E’ in Figure 12a. Table 2 summarizes the performance parameters for the bimetallic structure profiles labelled ‘A’ to ‘E’ in Figure 9. In Table 2, it can be seen that the sensitivity performance for the bimetallic layer was slightly worse than the uniform gold sensor case, which was explained in the earlier section. On the other hand, the *FWHM* of the bimetallic structure was 1.6 times narrower than the uniform gold sensor case. This led to a 1.34 times improvement in *FoM* of the bimetallic layer compared to the uniform gold sensor case. The bimetallic layer could not enhance the voltage sensing performance much in angular SPR measurement.

#### 3.2.2. Measurement Mechanism 2 (*M2*)

**Figure 13:**
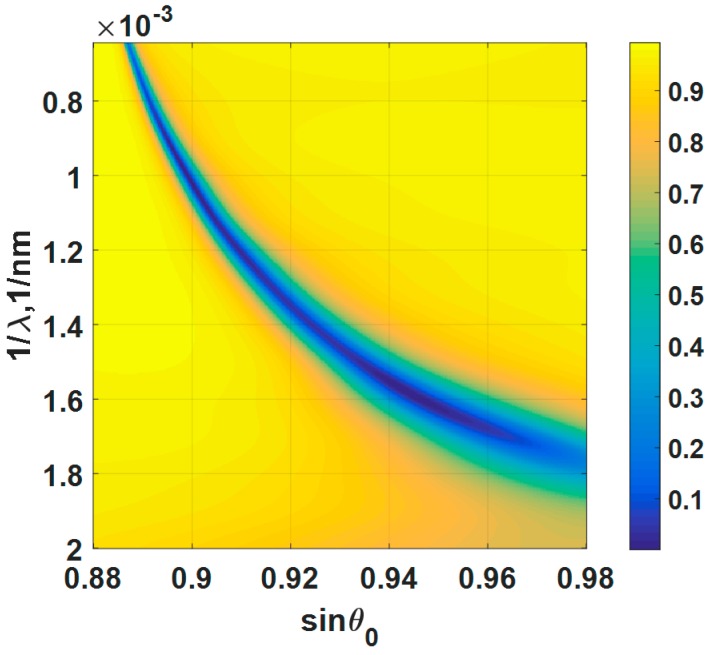
Reflectance spectrum of different incident angles and incident wavelengths, when the bimetallic layer consisting of a silver layer 31 nm thick and a gold layer 15 nm thick with the external applied potential *ψ*_0_ of 0 V.

**Figure 14:**
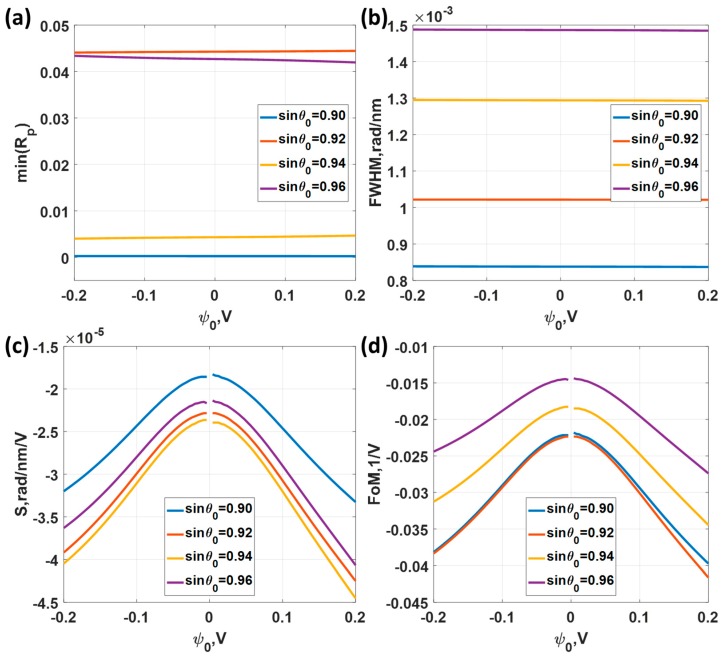
Responses when the bimetallic layer consisting of a silver layer 31 nm thick and a gold layer 15 nm thick for different applied potential levels ranging from −0.2 V to 0.2 V and the incident angles of sin*θ*_0_ of 0.90, 0.92, 0.94 and 0.96. (**a**) Minimum reflectance intensity level at the plasmonic wavelengths min(*R_p_*); (**b**) full width half maximum; (**c**) Sensitivity; and (**d**) Figure of Merit.

In this section, we took the structure profile that gave the best performance parameter from *M1* section, which is operating point ‘A’: *d*_1_ of 31 nm and *d*_2_ of 15 nm. Figure 13 shows *R_p_* responses for different incident wavelengths and incident angles. To compare the performance of the bimetallic layered structure and the uniform gold layer in wavelength scanning, the same range of the incident angle sin*θ*_0_ of 0.90, 0.92, 0.94 and 0.96 were chosen to calculate the performance parameters as shown in Figure 14. The minimum reflectance at the plasmonic dips were well below 5% for all the cases as shown in Figure 14a. The *FWHM* was narrower for lower incident angle as shown in Figure 14b. The *FWHM* for the uniform gold case (see Table 1 measurement 2 for comparison) was two times wider than the bimetallic structure. The sensitivity was also better than the uniform gold case as shown in Figure 14c. This lead to a factor of two enhancement in *FoM* in wavelength scanning mechanism for the bimetallic structure compared to the uniform gold sample. Table 3 summarizes the performance of the four operating points discussed in this section. This proposed procedure has allowed us to determine what kind of detection mechanism should be implemented for a particular SPR sensing structure. Otherwise, it would not be possible to predict the performance across platform.

#### 3.2.3. Measurement Mechanism 3 (*M3*)

**Figure 15:**
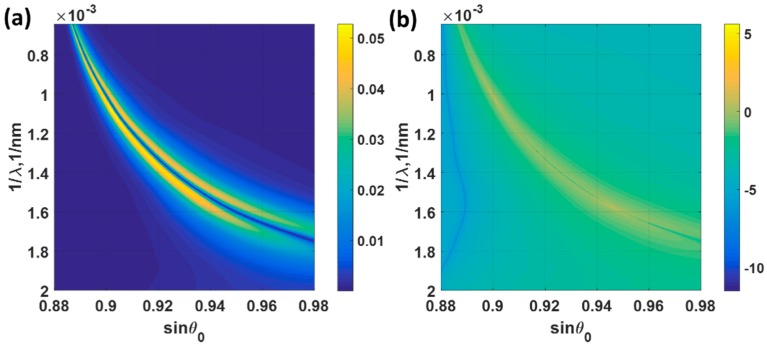
(**a**) Sensitivity (*S*) for mechanism 3 and (**b**) *FoM* calculated using Equation (8) with the structure profile of the operating point ‘B’ *d*_1_ of 25 nm and *d*_2_ of 20 nm. The sensitivity and *FoM* were calculated from *R_p_* responses of the biased voltage *ψ*_0_ of 0 V to 0.2 V.

The *FoM* for the Measurement mechanism 3 depends on two factors, which are the sensitivity and min(*R_p_*). From Table 2, the lowest min(*R_p_*) value was found in the operating point ‘B’, which was the *d*_1_ of 25 nm and *d*_2_ of 20 nm case followed by the operating point ‘A’ with the *d*_1_ of 31 nm and *d*_2_ of the 15 nm case. To calculate the sensitivity and the *FoM*, *R_p_* responses for the incident angles sin*θ*_0_ of 0.88 to 0.98 and incident wavelengths ranging from 500 nm to 1550 nm were simulated with the applied potentials *ψ*_0_ of 0 V and 0.2 V for the operating points ‘A’ to ‘D’. The performance parameters are summarized in Table 4. Only the results for operating point ‘B’ are shown in Figure 15. The operating point ‘B’ yielded the best sensitivity and *FoM* performance compared to the other operating points. The operating point B had the lowest min(*R_p_*) leading to the enhanced *FoM* of 0.6246, which was 25 times higher than the uniform gold sensor case.

It can be seen from this section that the bimetallic layer could not enhance the *FoM* for all the detection mechanisms. Mechanism 3 had the highest enhancement in the *FoM* due to the light intensity absorption mechanism of the bimetallic layer being higher than the single gold layer. Mechanism 2 had a higher *FoM* enhancement than mechanism 1. This gave us some insights into the SPR behavior that the bimetallic layer enhanced the SPR properties in wavelength domain not the angular domain. It is important to note that without the proposed calculation this would not be obvious to conclude.

## 4. Conclusions

There are several issues obscuring a direct performance comparison between SPR sensing structures for voltage sensing. Firstly, the electrolytic solution employed in each of the structures was different. Secondly, the SPR measurement mechanisms were different; and thirdly, of course, the sensor structures themselves were also different. In this paper, we have developed a theoretical framework enabling a direct comparison across different SPR platforms based on several performance parameters. We have employed the model developed by Abayzeed et al. and developed it further by defining performance parameters based on wave-vector and sensitivity per input power (*S*/*R_p_*) and figure of merit (*FoM*). We demonstrated the proposed concept by analyzing two SPR structures with three SPR detection mechanisms. The analyzed structures were the uniform gold layer and the bimetallic layer consisting of a uniform silver layer coated by another uniform gold layer. The SPR detection mechanisms were *M1* to measure the change in the plasmonic coupling angle, *M2* to measure the change in the plasmonic coupling wavelength and *M3* to measure the change in SP intensity level when the incident angle and incident wavelength were fixed.

For the uniform gold sensor with the optimum thickness of 47 nm, method *M2* had a higher sensitivity compared to method *M1*. The *FWHM*, however, had an opposite trend. The uniform gold sensor used in method *M1* had a narrower *FWHM* than method *M2*. These opposite effects therefore cancelled each other out in the *FoM* calculation. The uniform gold used in method *M1* had a slightly better performance than method *M2*. For method *M3*, we have discussed the sensitivity per input power and proposed a procedure to normalize the sensitivity per input power to compare with method *M1* and *M2*. The detection mechanisms and the SPR sensor structures should be chosen based on the performance parameters. The sensitivity per input power should be considered for the detection mechanism 3 case without the phase detection technique. On the other hand, the *FoM* should be considered for detection mechanisms 1 and 2. Note that for detection mechanisms 1 and 2 with phase detection, the sensitivity is more important in these cases.

For the bimetallic layered structure, we have optimized the bimetallic structures for the three SPR measurement mechanisms. This structure did not show much performance improvement in method *M1*. The optimized structure consisting of 31 nm thick and gold layer of 15 nm thick can only enhance the *FoM* by 1.34 compared to the uniform gold sensor. On the other hand, method *M2* had a better performance than method *M1* in terms of the sensitivity, the *FWHM* and the *FoM*. In comparison with the uniform gold sensor, the bimetallic sensor had a factor of two improvement in the *FoM*. For method *M3*, the optimum thickness of 25.5 nm of silver and 20.0 nm of gold can lead to 25 times improvement in the *FoM* compared to the uniform gold sensor case.

This analysis has enabled us to not only compare the performance of the different SPR structure and SPR measurement mechanisms, but also to determine which detection mechanism should be implemented to achieve the best performance possible.

## Figures and Tables

**Table 1:** Summarizes the performance parameters for 47 nm thick uniform gold SPR sensors.

**SPR Measurement Mechanism 1**					
Incident wavelength λ (nm)	Plasmonic angle sin*θ*_sp_	Min(*R_p_*)	*FWHM* (rad/nm)	Sensitivity (*S*) rad/nm/V	Figure of Merit (*FoM*) 1/V
632.8	0.9522	1.10 × 10^−6^	4.75 × 10^−4^	1.16 × 10^−5^	0.0244
**SPR Measurement Mechanism 2**					
Plasmonic wavelength *λ_SP_* (nm)	Incident angle sin*θ*_0_	Min(*R_p_*)	*FWHM* (rad/nm)	Sensitivity (*S*) rad/nm/V	Figure of Merit (*FoM*) 1/V
1008.0	0.9000	7.99 × 10^−3^	15.59 ×10^−4^	3.37 × 10^−5^	0.0216
766.7	0.9200	27.24 × 10^−3^	18.84 ×10^−4^	4.24 × 10^−5^	0.0230
665.9	0.9400	32.61 × 10^−3^	21.26 ×10^−4^	4.51 × 10^−5^	0.0212
615.5	0.9600	9.35 × 10^−3^	23.26 ×10^−4^	3.86 × 10^−5^	0.0166
**SPR Measurement Mechanism 3**					
Incident wavelength λ (nm)	Incident angle sin*θ*_0_	*R_p_*	Sensitivity (*S*) 1/V	*S*/*R_p_*	Figure of Merit (*FoM*) 1/V
941.2	0.9034	6.14 × 10^−8^	9.52 × 10^−4^	15,500	0.0244

**Table 2:** Summarizes the performance parameters bimetallic layered structures for angular measurement mechanism.

SPR Measurement Mechanism 1								
Operating point	Silver thickness (*d*_1_) nm	Gold thickness (*d*_2_) nm	Incident wavelength *λ* (nm)	Plasmonic angle sin*θ*_sp_	Min(*R_p_*)	*FWHM* rad/nm	Sensitivity (*S*) rad/nm/V	Figure of Merit (*FoM*) 1/V
‘A’	31.0	15.0	632.8	0.9435	2.84 × 10^−7^	3.02 × 10^−4^	1.00 × 10^−5^	0.0333
‘B’	25.5	20.0	632.8	0.9464	8.33 × 10^−8^	3.50 × 10^−4^	1.06 × 10^−5^	0.0303
‘C’	20.5	25.0	632.8	0.9485	1.52 × 10^−6^	3.86 × 10^−4^	1.10 × 10^−5^	0.0285
‘D’	15.8	30.0	632.8	0.9500	2.03 × 10^−6^	4.11 × 10^−4^	1.12 × 10^−5^	0.0273
‘E’	38.2	15.0	632.8	0.9509	0.1	2.11 × 10^−4^	1.03 × 10^−5^	0.0488

**Table 3:** Summarizes the performance parameters bimetallic layered structure with *d*_1_ of 31 nm and *d*_2_ of 15 nm for plasmonic wavelength measurement.

SPR Measurement Mechanism 2							
Silver thickness (*d*_1_) nm	Gold thickness (*d*_2_) nm	Plasmonic wavelength *λ_SP_* (nm)	Incident angle sin*θ*_0_	Min(*R_p_*)	*FWHM* rad/nm	Sensitivity (*S*) rad/nm/V	Figure of Merit (*FoM*) 1/V
31.0	15.0	981.4	0.90	2.38 × 10^−4^	8.37 × 10^−4^	3.33 × 10^−5^	0.0397
31.0	15.0	740.7	0.92	4.44 × 10^−2^	10.21 × 10^−4^	4.25 × 10^−5^	0.0416
31.0	15.0	643.9	0.94	4.45 × 10^−3^	12.39 × 10^−4^	4.45 × 10^−5^	0.0347
31.0	15.0	595.2	0.96	4.28 × 10^−2^	14.85 × 10^−4^	4.07 × 10^−5^	0.0274

**Table 4:** Summarizes the performance parameters bimetallic layered structures with different *d*_1_ and *d*_2_ for mechanism 3 measurement.

SPR Measurement Mechanism 3								
Operating point	Silver thickness (*d*_1_) nm	Gold thickness (*d*_2_) nm	Incident wavelength λ(nm)	Incident angle sin*θ*_0_	*R_p_*	Sensitivity (*S*) 1/V	*S*/*R_p_*	Figure of Merit (*FoM*) 1/V
‘A’	31.0	15.0	994.5	0.8994	4.30 × 10^−7^	14.23 × 10^−4^	3306	0.0052
‘B’	25.5	20.0	632.9	0.9464	1.89 × 10^−9^	7.48 × 10^−4^	396,777	0.6246
‘C’	20.5	25.0	965.9	0.9016	1.79 × 10^−8^	9.04 × 10^−4^	50,556	0.0796
‘D’	15.8	30.0	814.5	0.9130	9.96 × 10^−7^	10.86 × 10^−4^	1,090	0.0017
